# Novel Fatty Acid Chain-Shortening by Fungal Peroxygenases Yielding 2C-Shorter Dicarboxylic Acids

**DOI:** 10.3390/antiox11040744

**Published:** 2022-04-08

**Authors:** Andrés Olmedo, René Ullrich, Martin Hofrichter, José C. del Río, Ángel T. Martínez, Ana Gutiérrez

**Affiliations:** 1 Instituto de Recursos Naturales y Agrobiología de Sevilla, CSIC, Av. Reina Mercedes 10, 41012 Seville, Spain; aolmedo@irnas.csic.es (A.O.); delrio@irnase.csic.es (J.C.d.R.); 2 Unit of Bio- and Environmental Sciences, TU Dresden, International Institute Zittau, Markt 23, 02763 Zittau, Germany; rene.ullrich@tu-dresden.de (R.U.); martin.hofrichter@tu-dresden.de (M.H.); 3 Centro de Investigaciones Biológicas “Margarita Salas”, CSIC, Ramiro de Maeztu 9, 28040 Madrid, Spain; atmartinez@cib.csic.es

**Keywords:** chain shortening, fatty acids, peroxygenation, EC 1.11.2.1, *Coprinopsis cinerea*, *Agrocybe aegerita*, fungal enzymes

## Abstract

Unspecific peroxygenases (UPOs), the extracellular enzymes capable of oxygenating a potpourri of aliphatic and aromatic substrates with a peroxide as co-substrate, come out with a new reaction: carbon-chain shortening during the conversion of fatty acids with the well-known UPOs from *Coprinopsis cinerea* (r*Cci*UPO) and *Cyclocybe* (*Agrocybe*) *aegerita* (*Aae*UPO). Although a pathway (Cα-oxidation) for shortening the hydrocarbon chain of saturated fatty acids has already been reported for the UPO from *Marasmius rotula* (*Mro*UPO), it turned out that r*Cci*UPO and *Aae*UPO shorten the chain length of both saturated and unsaturated fatty acids in a different way. Thus, the reaction sequence does not necessarily start at the Cα-carbon (adjacent to the carboxyl group), as in the case of *Mro*UPO, but proceeds through the subterminal (ω-1 and ω-2) carbons of the chain via several oxygenations. This new type of shortening leads to the formation of a dicarboxylic fatty acid reduced in size by two carbon atoms in the first step, which can subsequently be further shortened, carbon by carbon, by the UPO Cα-oxidation mechanism.

## 1. Introduction

In recent years (2004 till present), progress has been made in the study of a novel group of biocatalysts with peroxygenating activity (i.e., transfer of a peroxide-borne oxygen atom to substrates), currently referred to as unspecific peroxygenases (UPO, EC 1.11.2.1) [1]. Most of them are extracellular heme-thiolate proteins secreted by fungi, and only require the presence of a peroxide to perform oxygen transfers. Beyond the subsequent advantages in terms of stability and lack of auxiliary enzymes/modules and sources of reducing power, they have shown to catalyze a wide range of oxyfunctionalization reactions, including the oxygenation of aromatic compounds, alkanes, alkenes, alcohols, fatty acids, heteroatoms and steroids, as well as *N*-/*O*-dealkylations, among other reactions [1].

Since the discovery of the first enzyme of this type in the agaric fungus *Cyclocybe* (*Agrocybe) aegerita* (*Aae*UPO), first described as a haloperoxidase [2] (that, for example, brominates various organic substrates), the number of reactions catalyzed has been steadily increased. In addition to halogenation, it was shown to transfer oxygen to aromatic compounds [3], which is why it was classified—at the time—as an aromatic peroxygenase. Finally, once its ability to catalyze the hydroxylation of diverse aliphatic compounds had been demonstrated [4,5], it was given the ultimate name unspecific peroxygenase (UPO). Following *Aae*UPO, more UPOs were discovered in other fungal species such as *Coprinellus radians*, which showed similarities to *Aae*UPO in terms of substrates and reaction conditions [6], or differed from it, such as the enzyme from *Marasmius rotula* (*Mro*UPO), the first UPO with pronounced activity towards bulky substrates [7]. Thereafter, a recombinant UPO from the fungus *Coprinopsis cinerea* (r*Cci*UPO) became available, showing some analogies to *Aae*UPO regarding aliphatic oxygenation [8]. Since then, several more UPOs have been characterized, either wild-types as those from *Marasmius wettsteinii* or *Chaetomium globosum (Mwe*UPO and *Cgl*UPO), or recombinant proteins from *Humicola insolens*, *Collariella virescens* and *Daldinia caldariorum* (r*Hin*UPO, r*Cvi*UPO and r*Dca*UPO), among others [9,10].

Most of the aforementioned oxyfunctionalization reactions had been attributed to P450 monooxygenases (P450s) until UPOs appeared on the “biochemical scene”. Among the potpourri of reactions catalyzed by UPOs, there is a particular one, first described for *Mro*UPO in 2017 and not accomplished by P450s, namely the shortening of the saturated hydrocarbon chain (carbon by carbon) of either mono- or dicarboxylic fatty acids [11]. This chain-shortening reaction starts with the enzymatic hydroxylation of the carbon adjacent to the carboxylic group, the α-carbon, which is subsequently oxygenated again by the enzyme, giving an α-keto intermediate, which is in turn chemically decarboxylated by hydrogen peroxide present in the reaction to yield a carboxylic fatty acid reduced in size by one carbon atom. Just after the publication of these findings, a similar reaction was reported to be catalyzed by a P450 enzyme [12]. Moreover, at that time, another shortening reaction was observed during the side-chain removal from certain steroids by *Mro*UPO and *Mwe*UPO [13].

In the present work, we report on a novel chain-shortening reaction (initial reduction in size by two carbon atoms) during the conversion of saturated and unsaturated fatty acids catalyzed by r*Cci*UPO and *Aae*UPO.

## 2. Materials and Methods

### 2.1. Enzymes

*Aae*UPO is a wild-type enzyme obtained from cultures of the agaric basidiomycete *A. aegerita* TM-A1 in soybean-peptone medium, and purified as described elsewhere [3]. Recombinant r*Cci*UPO was supplied by Novozymes A/S and corresponds to the protein model 7249 from the genome of *C. cinerea* sequenced at the DOE JGI (http://genome.jgi.doe.gov/Copci1; accessed on 1 February 2022) expressed in *A. oryzae* (patent WO/2008/119780). Enzyme concentrations were spectrophotometrically estimated based on the spectra of the reduced-UPO adducts with carbon monoxide (CO) [14].

### 2.2. Model Substrates

A series of fatty acids, including both saturated—such as myristic (C14:0), palmitic (C16:0) and stearic (C18:0) acids—and unsaturated ones—such as myristoleic (C14:1), palmitoleic (C16:1), oleic (C18:1), linoleic (C18:2) and α-linolenic (C18:3) acids—(from Sigma-Aldrich, San Luis, MI, USA) were used as substrates of r*Cci*UPO and *Aae*UPO.

### 2.3. Enzymatic Reactions

Reactions of fatty acids (0.1 mM) with r*Cci*UPO and *Aae*UPO were performed in 2-mL vials containing 50 mM sodium phosphate buffer (pH 7) at 30 °C, in the presence of H_2_O_2_ (2.5–20 mM) over 0.5–25 h. The enzyme concentration varied from 0.2 to 1.4 μM in 20% or 40% of acetone in a total volume of 1 mL. A time course experiment was also performed under these conditions, but using 2.5 mM of H_2_O_2_ and an enzyme concentration of 0.2 μM for 2, 4, 8 and 12 min. Likewise, reactions in the absence of any enzyme (2 h, 1–15 mM H_2_O_2_), as well as in the absence of peroxide (2 h, 0.2–2 μM r*Cci*UPO), were performed within the mentioned concentration ranges.

Products were recovered by liquid-liquid extraction with methyl *tert*-butyl ether and dried under N_2_ flow. *N*,*O*-Bis(trimethylsilyl)trifluoroacetamide (Supelco, Bellefonte, PA, USA) was used to prepare trimethylsilyl (TMS) derivatives that were analyzed by gas chromatography-mass spectrometry (GC-MS), as described below.

### 2.4. GC-MS Analyses

The analyses were performed with a Shimadzu GC-MS QP2010 Ultra, using a fused-silica DB-5HT capillary column (30 m × 0.25 mm internal diameter, 0.1 μm film thickness) from J&W Scientific (Folsom, CA, USA). The oven was heated from 120 °C (1 min) to 300 °C (15 min) at 5 °C·min^−1^. The injection was performed at 300 °C, and the transfer line was kept at 300 °C. Compounds were identified by mass fragmentography, and by comparing their mass spectra with those of the Wiley and NIST libraries and standards. Quantification was obtained from total-ion peak area, using molar response factors of the same or similar compounds.

## 3. Results

**Scheme 1:**
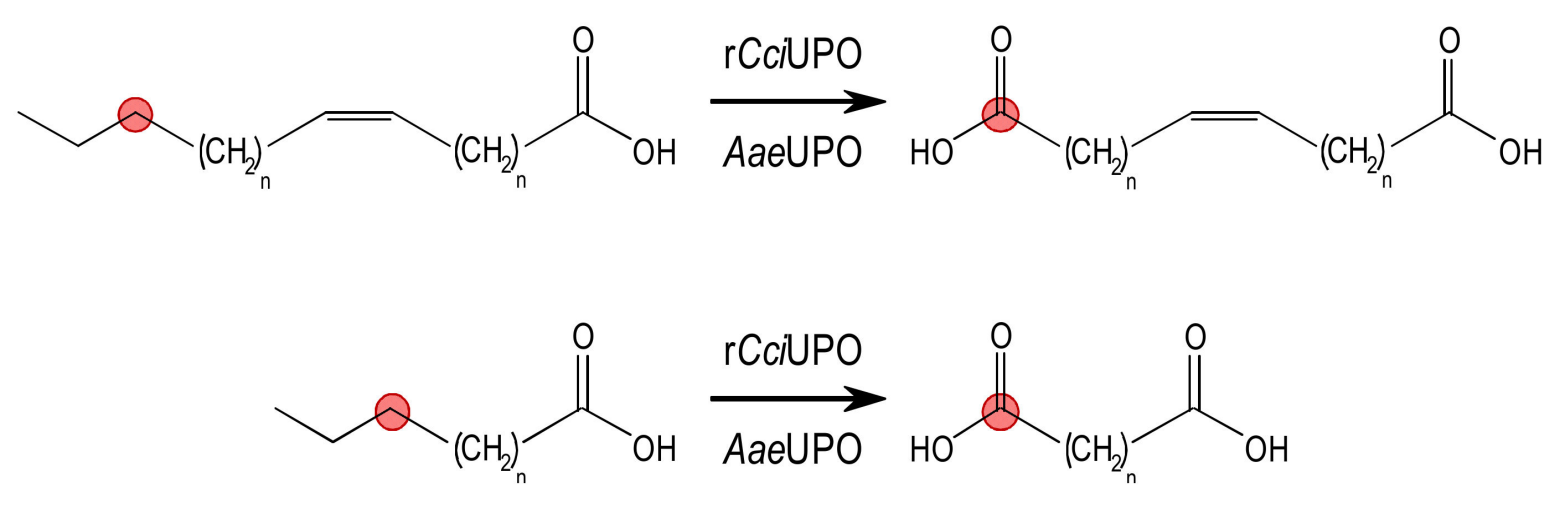
Saturated and unsaturated monocarboxylic fatty acids (**left**) are shortened by two unspecific peroxygenases (UPOs) yielding 2C-shorter alkyl-chain dicarboxylic acids (**right**).

This work reports a new reaction catalyzed by the first described wild-type fungal peroxygenase (*Aae*UPO) and the first recombinant peroxygenase (r*Cci*UPO). The two enzymes were previously reported to hydroxylate fatty acids, both the (mono- and di-) unsaturated and saturated ones, at subterminal positions forming the corresponding oxygenated (hydroxy and keto) derivatives at ω-1 and ω-2 carbons [5,8]. Aimed at studying these reactions more in depth (under different conditions), it was found that these enzymes are able to catalyze a shortening reaction of the fatty acid alkyl chains yielding 2C-shorter alkyl-chain dicarboxylic acids (Scheme 1), as described below.

### 3.1. Fatty Acid Chain-Shortening by rCciUPO and AaeUPO

**Figure 1:**
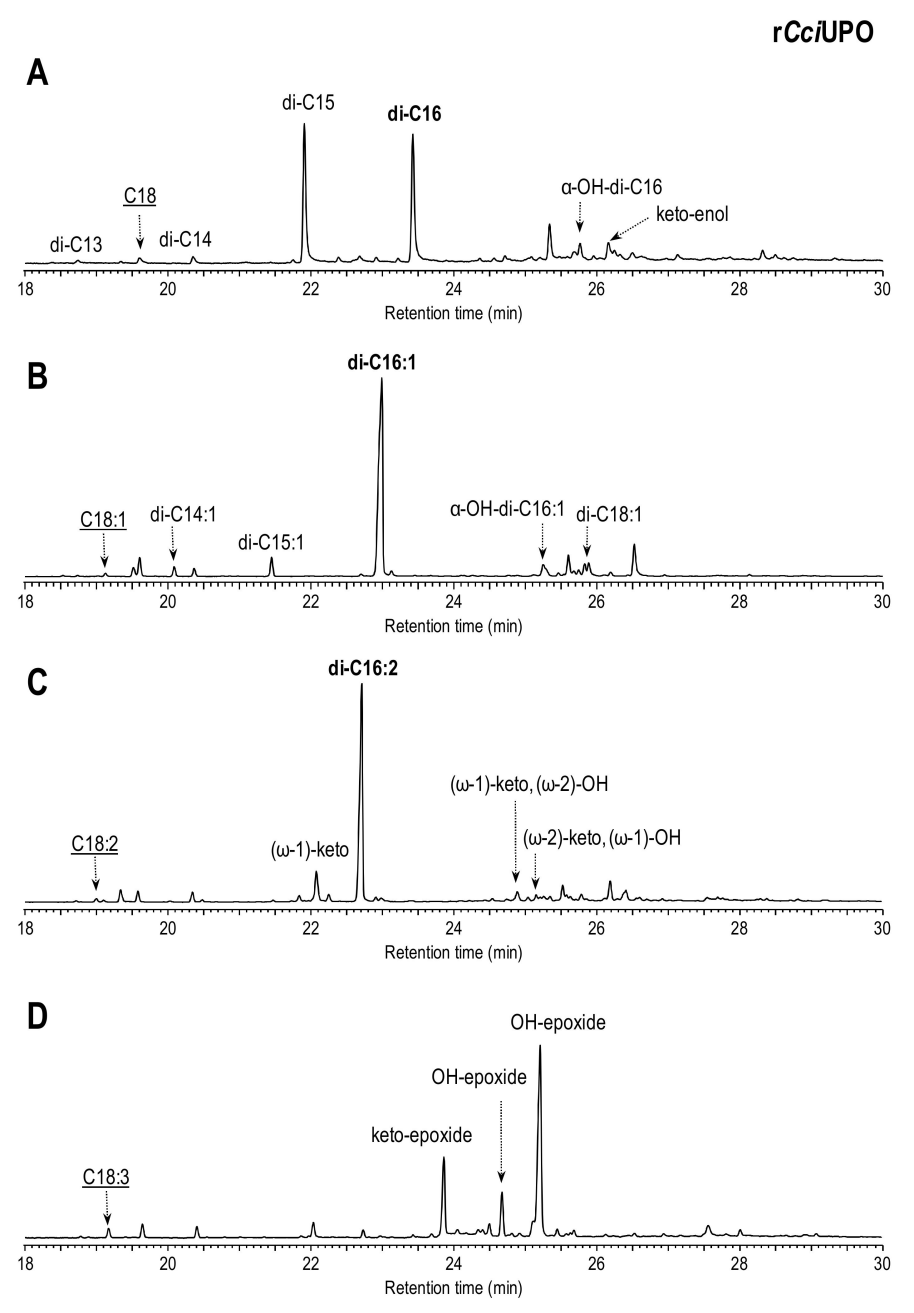
Gas chromatography-mass spectrometry (GC-MS) analysis of r*Cci*UPO reactions with 0.1 mM stearic (**A**), oleic (**B**), linoleic (**C**) and linolenic (**D**) acids (underlined), showing the dicarboxylic acids (in bold), together with the hydroxy, keto, enol and epoxide derivatives. Reaction conditions: A (1.4 μM enzyme, 20 mM H_2_O_2_, 25 h) and B, C, D (0.2 μM enzyme, 2.5 mM H_2_O_2_, 0.5 h).

A series of fatty acids, including both saturated and unsaturated ones, namely myristic (C14), myristoleic (C14:1), palmitic (C16), palmitoleic (C16:1), stearic (C18), oleic (C18:1), linoleic (C18:2) and α-linolenic (C18:3) acids, were selected as substrates of r*Cci*UPO and *Aae*UPO. With the purpose of investigating how their reactions would proceed after the formation of primary (monohydroxylated) reaction products, higher enzyme and/or peroxide loadings with respect to previous works [5,8] were used. Surprisingly, in the course of the experiments, GC-MS analyses of r*Cci*UPO reactions revealed that with all substrates, except for myristoleic and α-linolenic acids, a new product appeared that was consistent with a dicarboxylic acid having a 2C-shorter alkyl-chain than the corresponding substrate (Figure 1 and Figure S1, Scheme 1). In general, these shortening reactions were seemingly more efficient with unsaturated fatty acids such as oleic and linoleic acids (Figure 1B,C), forming di-C16:1 and diC16:2, respectively, followed by palmitoleic acid (Figure S1D) forming di-C14:1, than with the saturated counterparts, such as stearic, palmitic and myristic acids (Figure 1A and Figure S1A,C), forming di-C16, di-C14 and di-C12, respectively, since stronger reaction conditions were required with the latter to obtain the likewise shortened fatty acids.

With myristoleic acid, the predominant products were the monooxygenated (hydroxy and keto) derivatives at the ω-1 and ω-2 positions (Figure S1B), and with α-linolenic acid, the main products were the monooxygenated (hydroxy and keto) derivatives of the epoxide (at the last double bond) (Figure 1D), as previously reported [15], but no shortened products were observed for neither of them. Likewise, the products from *Aae*UPO reactions with these fatty acids were similar to those of r*Cci*UPO, and included the corresponding 2C-shorter dicarboxylic acids (Figures S2 and S3), although they were generally produced less efficiently. Additionally, like r*Cci*UPO, *Aae*UPO failed to shorten myristoleic (Figure S3B) and α-linolenic (Figure S2D) acids that were mainly converted to the monooxygenated derivatives at the subterminal (ω-1 and ω-2) positions, the former, and to the epoxide (at the last double bond), the latter. Other UPOs, such as *Mro*UPO, *Cgl*UPO, r*Hin*UPO and r*Dca*UPO were also tested with these fatty acids under the same conditions, but failed to give this shortening reaction (data not shown).

### 3.2. Chain-Shortening Mechanism

**Figure 2:**
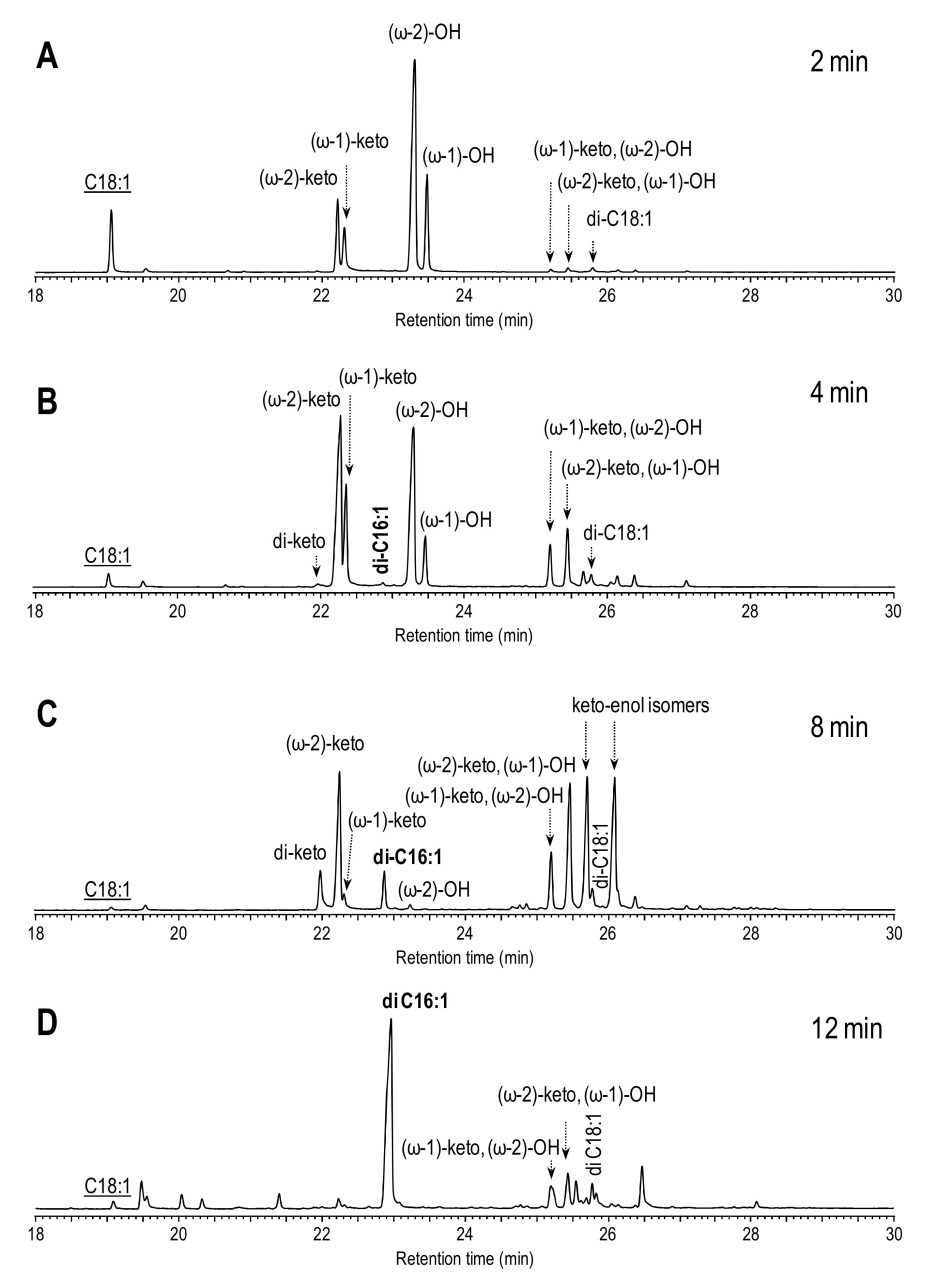
GC-MS evaluation of a time-course experiment with 0.2 μM r*Cci*UPO and 0.1 mM oleic acid (underlined), within 2 min (0.5 mM H_2_O_2_) (**A**), 4 min (1 mM H_2_O_2_) (**B**), 8 min (2 mM H_2_O_2_) (**C**) and 12 min (3 mM H_2_O_2_) (**D**), showing the dicarboxylic acids (in bold), together with the hydroxy, with minor keto, enol and epoxide derivatives. The chemical structures of oleic acid and their derivatives are depicted in Figure 3.

**Figure 3:**
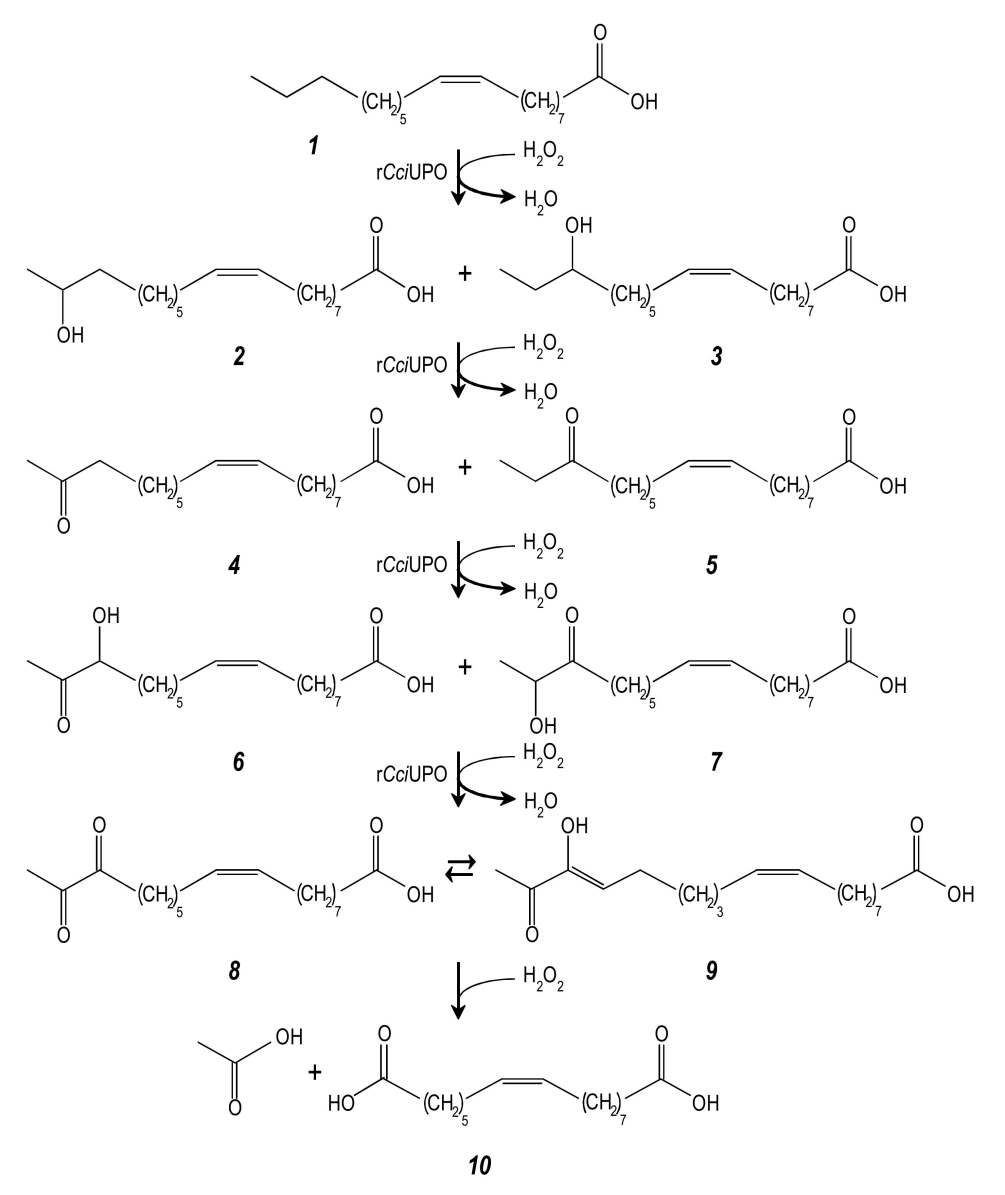
Proposed pathway for the 2C-shortening of fatty acids by UPOs (and their H_2_O_2_ cosubstrate) showing the structures of oleic acid (**1**), its monohydroxylated derivatives at ω-1 (**2**) and ω-2 (**3**) positions, the mono-keto derivatives at ω-1 (**4**) and ω-2 (**5**) positions, the dioxygenated hydroxy-keto (**6**) and keto-hydroxy (**7**) derivatives, the di-keto (**8**), in equilibrium with its keto-enol isomer (**9**) derivatives, and finally the 2C shortened dicarboxylic acid (**10**) from H_2_O_2_ cleavage of the diketo C-C bond.

Aiming at finding out the mechanism of this new 2C-shortening reaction, a time-course experiment with r*Cci*UPO and one particular fatty acid (oleic acid) was performed and evaluated by GC-MS (Figure 2, chemical structures in Figure 3). The main products of oleic acid (**1**, Figure 3) within the first 2 min of reaction (Figure 2A) were the mono-hydroxylated derivatives of oleic acid at ω-1 (**2**) and ω-2 (**3**) positions along with minor amounts of the corresponding keto derivatives at these subterminal positions (**4** and **5**, respectively).

Within 4 min of reaction (Figure 2B), the amount of ω-1 and ω-2 keto derivatives increased, and dioxygenated derivatives, namely (ω-1)-keto, (ω-2)-hydroxy oleic acid (**6**) and (ω-1)-hydroxy, (ω-2)-keto oleic acid (**7**) became apparent. Additionally, traces of a new peak corresponding to 16 C-atoms emerged, namely di-C16:1 (**10**), together with traces of other derivatives, such as di-keto compounds (**8**) and dicarboxylic (di-C18:1) acids. Within 8 min (Figure 2C), the amount of di-keto, di-C16:1 and dioxygenated derivatives increased, and two new peaks identified as the keto-enol (**9**) isomers of these dioxygenated compounds appeared. Finally, within 12 min, the predominant product of oleic acid oxidation was the di-C16:1 (Figure 2D). Based on these findings, the C-chain shortening may follow the pathway illustrated in Figure 3.

**Figure 4:**
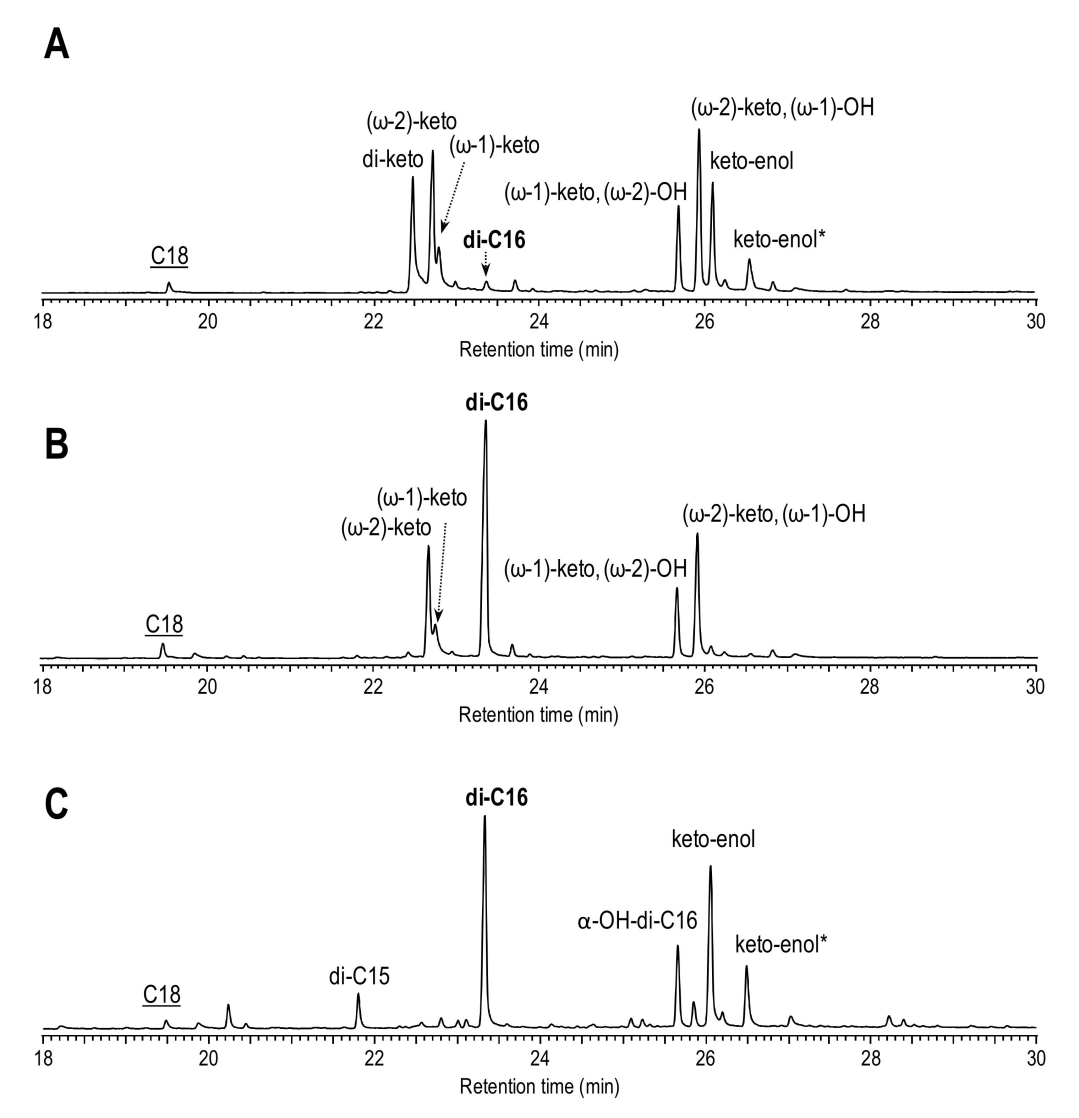
GC-MS profiles of the reaction of 1.2 μM r*Cci*UPO with 0.1 mM stearic acid (underlined), after 3 h and 10 mM H_2_O_2_ (**A**), extracted and used as substrate of reactions B and C; the products of the reaction A after addition of 15 mM H_2_O_2_ during 2 h in the absence of any enzyme (**B**), and the products of A with 1 μM r*Cci*UPO after 2 h and 15 mM H_2_O_2_ (**C**). The solvent acetone was added in all cases (40% vol/vol). The 2C-shorter dicarboxylic acid (in bold) is shown together with the hydroxy, keto and enol derivatives. Peaks with asterisk were tentatively identified as keto-enol isomers.

Trying to ascertain the shortening mechanism, and particularly to decipher the last step, in which the di-keto and keto-enol isomers disappear completely, and the shortened fatty acid becomes predominant, a couple of reactions in the absence and presence of r*Cci*UPO were carried out at a time, using the products of a previous reaction of stearic acid (C18) with r*Cci*UPO as substrate (Figure 4). The reaction mixture used as the substrate of this new reaction was selected due to its high content of the di-keto C18 and keto-enol C18 isomers (Figure 4A). The reaction without enzyme, hence with the addition of hydrogen peroxide only, showed a conversion of both mentioned products into the shortened dicarboxylic acid (di-C16) (Figure 4B). The assay with r*Cci*UPO revealed no mono-oxygenated derivatives among the products, besides the keto-enol derivatives and the oxygenation of the di-carboxylic acid (α-OH di-C16) and its again shortened di-C15 (Figure 4C). Next, reactions of this substrate with several H_2_O_2_ concentrations were performed that revealed a concomitant conversion of di-keto and keto-enol derivatives into the shortened product with increasing H_2_O_2_ concentrations (Figure S4). Finally, reactions with different r*Cci*UPO doses (in the absence of H_2_O_2_) were carried out, showing no conversion of the di-keto and keto-enol derivatives into the shortened product (data not shown).

## 4. Discussion

We report here a new chain-shortening reaction that was first observed during the conversion of oleic acid with r*Cci*UPO and *Aae*UPO, where several “unfamiliar” chromatographic peaks appeared among the previously known products. Once the two-carbon shortened di-carboxylic acid (hexadecenedioic acid, di-C16:1) was identified in the reaction of oleic acid, our first hypothesis (A) was directed to the already described chain shortening mechanism by *Mro*UPO [11]. In the course of this reaction, the starting compound would be the *cis*-9-octadecenedioic acid (di-C18:1, observed among the products), which would be shortened carbon by carbon via α-hydroxy di-C18:1.

Aiming at getting insight into the shortening mechanism, we performed a time-course experiment of this reaction. The results obtained questioned the hypothesis A because of the low amount of di-C18:1 observed along the whole time-course experiment, together with the absence of both α-hydroxy-di-C18:1 and di-C17:1. On the other hand, the presence of a high amount of di-oxygenated (hydroxy-keto, keto-enol and di-keto) derivatives at the subterminal positions (ω-1 and ω-2) led us to the hypothesis B. It considers a new kind of shortening reaction never described before for an UPO (or likewise P450), starting from the monocarboxylic acid (oleic acid) (Figure 3). The reaction may start with the hydroxylation of both (ω-1 and ω-2) subterminal carbons of the mono-carboxylic fatty acid, replaying the already known activity of r*Cci*UPO and *Aae*UPO. These monohydroxylated derivatives would be further oxygenated to give the vicinal diketones (identified together with keto-enol isomers that would be directly cleaved between the two carbonyl groups). This shortening reaction has been observed not only with oleic acid (C18:1), but also with other fatty acids like linoleic (C18:2), stearic (C18) and myristic (C14) acids, indicating its relevance for saturated fatty acids as well. On the other hand, this shortening was not observed with myristoleic or with α-linolenic acids, most probably because neither were converted to their corresponding vicinal diketones, which seems to be a necessary step for this shortening reaction. In the case of α-linolenic acid, this may be due to its preferential epoxidation [15].

Finally, enzymatic conversions using the product mixture of the reaction of r*Cci*UPO with stearic acid in the presence of peroxide (with and without enzyme) as a substrate confirmed that the oxidation of the di-keto derivatives by hydrogen peroxide involves the breaking of the carbon-carbon bond between the carbonyl groups, with formation of two carboxyl groups, as described previously [16]. The reaction may lead to the formation of the 2C-shorter di-carboxylic fatty acid, most probably by the release of acetic acid. This mechanism differs from that previously described for the Cα-oxidative decarboxylation of fatty acids by *Mro*UPO (α-oxidation), which only functioned with saturated (and not with unsaturated) fatty acids, yielding a one-carbon shorter-alkyl-chain fatty acid [11], and also from the possible peroxide shortening of corticosteroids by *Mro*UPO and *Mwe*UPO, in which a hydroperoxy derivative may be formed at the carbon adjacent to the carboxylic group, combining a tetrahedral intermediate which would decompose by releasing carbon dioxide and hence yielding the C1-shorter product [13]. In this reaction (Figure 4), r*Cci*UPO did not only convert mono-keto derivatives into di-oxygenated products (keto-enols) that generated di-C16, but also produced the shortened again di-C15 acid by Cα-oxygenation of the di-C16, as described for *Mro*UPO [11]. The shortening of saturated and unsaturated dicarboxylic acids, carbon by carbon, via α-oxidation, is reported here for the first time for r*Cci*UPO and *Aae*UPO.

Whether fatty acid shortening and other UPO reactions are of physiological/ecological relevance for fungi, independently of their recognized biotechnological importance, is still unclear. One can speculate that extracellular shortening/degradation of fatty acids, which are important signaling molecules in various organisms [17], could interrupt signaling cascades that, in turn, would lead to a competitive advantage of UPO-secreting fungi. A second explanation could be that the formation of acetic acid during extracellular shortening of fatty acids helps to establish a low pH in the hyphal microenvironment, which is preferred by most filamentous fungi colonizing leaf-litter, deadwood and related habitats [18].

## 5. Conclusions

Although the shortening by two carbons at a time is common in nature in the form of the β-oxidation of fatty acids in plants, fungi and animals (involving several steps and enzymes) [19], the reactions reported here are an interesting alternative that leads to the formation of di-carboxylic acids and starts from the opposite side of the chain that does not contain the activating carboxylic group. This pathway is directed by the enzyme’s own previous oxygenations, and therefore needs only one catalyst for the entire reaction sequence. This new shortening reaction does not only lead to the formation of a variety of tailor-made di-carboxylic acids, with the advantages of the odd-even effect [20], but also expands the chemical portfolio of oxyfunctionalization reactions performed by these extraordinary biocatalysts.

## Data Availability

All data underlying this article are available in the main publication and in its Supplementary Materials online.

## Supplementary Materials

The following supporting information can be downloaded at: https://www.mdpi.com/article/10.3390/antiox11040744/s1: Figure S1: GC-MS analysis of r*Cci*UPO reactions with myristic (A), myristoleic (B), palmitic (C) and palmitoleic (D) acids (underlined), showing the shortened dicarboxylic acids (in bold) together with the hydroxy, keto, and enol derivatives of the substrate. Reaction conditions: A (0.1 mM substrate, 0.2 μM enzyme, 2.5 mM H_2_O_2_, 0.5 h, 20% acetone); B (0.5 mM substrate, 1 μM enzyme, 20 mM H_2_O_2_, 24 h, 20% acetone); C (0.1 mM substrate, 0.8 μM enzyme, 15 mM H_2_O_2_, 3 h, 40% acetone); and D (0.1 mM substrate, 0.8 μM enzyme, 15 mM H_2_O_2_, 3 h, 20% acetone). The peaks with question marks are tentatively assigned as keto-enol isomers; Figure S2: GC-MS analysis of *Aae*UPO reactions with 0.1 mM stearic (A), oleic (B), linoleic (C) and linolenic (D) acids (underlined) showing the dicarboxylic acids (in bold) together with the hydroxy, keto, enol and epoxide derivatives. Reaction conditions: A (1 μM enzyme, 20 mM H_2_O_2_, 25 h); B (0.4 μM enzyme, 5 mM H_2_O_2_, 1 h) and C, D (0.2 μM enzyme, 2.5 mM H_2_O_2_, 0.5 h); Figure S3: GC-MS analysis of *Aae*UPO reactions with myristic (A), myristoleic (B), palmitic (C) and palmitoleic (D) acids (underlined) showing the shortened dicarboxylic acids (in bold) together with the hydroxy, keto, and enol derivatives of the substrate. Reaction conditions: A (0.1 mM substrate, 0.4 μM enzyme, 5 mM H_2_O_2_, 1 h, 20% acetone); B (0.1 mM substrate, 0.2 μM enzyme, 2.5 mM H_2_O_2_, 0.5 h, 20% acetone); C (0.1 mM substrate, 0.8 μM enzyme, 15 mM H_2_O_2_, 3 h, 40% acetone); and D (0.1 mM substrate, 0.8 μM enzyme, 15 mM H_2_O_2_, 3 h, 20% acetone); Figure S4: GC-MS of the reaction of 1.2 μM r*Cci*UPO with 0.1 mM stearic acid (underlined), after 3 h and 10 mM H_2_O_2_ (A). GC-MS of the products of the reaction A after addition of 1 mM (B), 3 mM (C) and 5 mM (D) H_2_O_2_ during 2 h in the absence of any enzyme. Higher doses of H_2_O_2_ (up to 15 mM) did not show differences with respect to D. Solvent (acetone) was added in a 40% of the total volume in all cases. The 2C-shorter dicarboxylic acid (in bold) is shown together with the hydroxy, keto and enol derivatives.
