# Supplementary material for: Novel Fatty Acid Chain-Shortening by Fungal Peroxygenases Yielding 2C-Shorter Dicarboxylic Acids
Source: Antioxidants (Basel). 2022 Apr 8;11(4):744. doi: 10.3390/antiox11040744 (PMC9025384; doi:10.3390/antiox11040744)
Supplement: Supplementary file 1 [file antioxidants-11-00744-s001.zip › antioxidants-1653515Supplementary.pdf]

These Supplementary Materials include the GC-MS analyses of the reactions by r*Cci*UPO with myristic, myristoleic, palmitic and palmitoleic acids (Figure S1); *Aae*UPO with stearic, oleic, linoleic and α-linolenic acids (Figure S2) and *Aae*UPO with myristic, myristoleic, palmitic and palmitoleic acids (Figure S3); and several doses of H_2O_2 (without enzyme) on the products of the reaction of r*Cci*UPO with stearic acid (Figure S4).

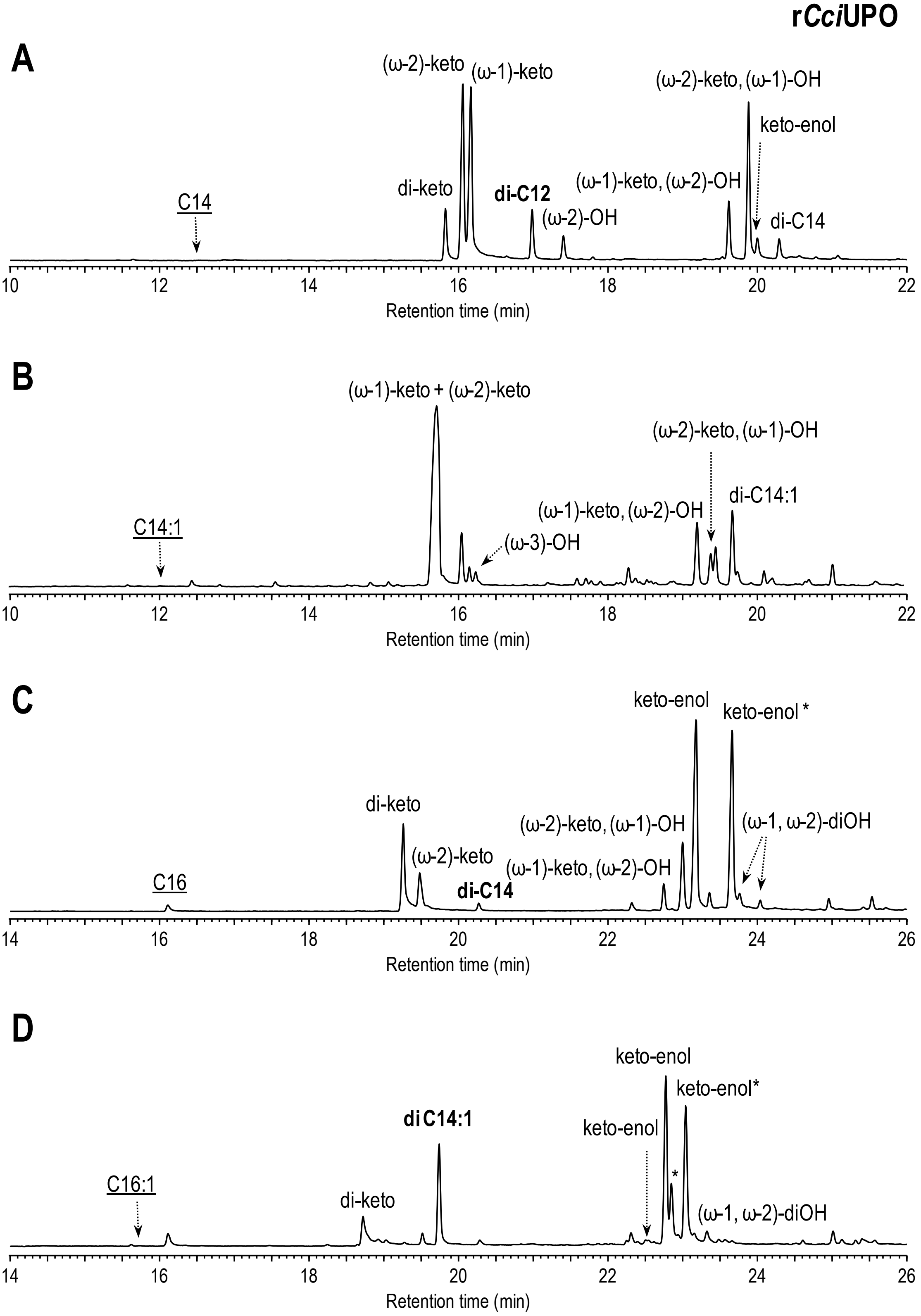

Figure S1. GC-MS analysis of r*Cci*UPO reactions with myristic (**A**), myristoleic (**B**), palmitic (**C**) and palmitoleic (**D**) acids (underlined) showing the shortened dicarboxylic acids (in bold) together with

the hydroxy, keto, and enol derivatives of the substrate. Reaction conditions: A (0.1 mM substrate, 0.2 μM enzyme, 2.5 mM H_2O_2, 0.5 h, 20% acetone); B (0.5 mM substrate, 1 μM enzyme, 20 mM H_2O_2, 24 h, 20% acetone); C (0.1 mM substrate, 0.8 μM enzyme, 15 mM H_2O_2, 3 h, 40% acetone) and D (0.1 mM substrate, 0.8 μM enzyme, 15 mM H_2O_2, 3 h, 20% acetone). Peaks with asterisk were tentatively identified as keto-enol isomers.

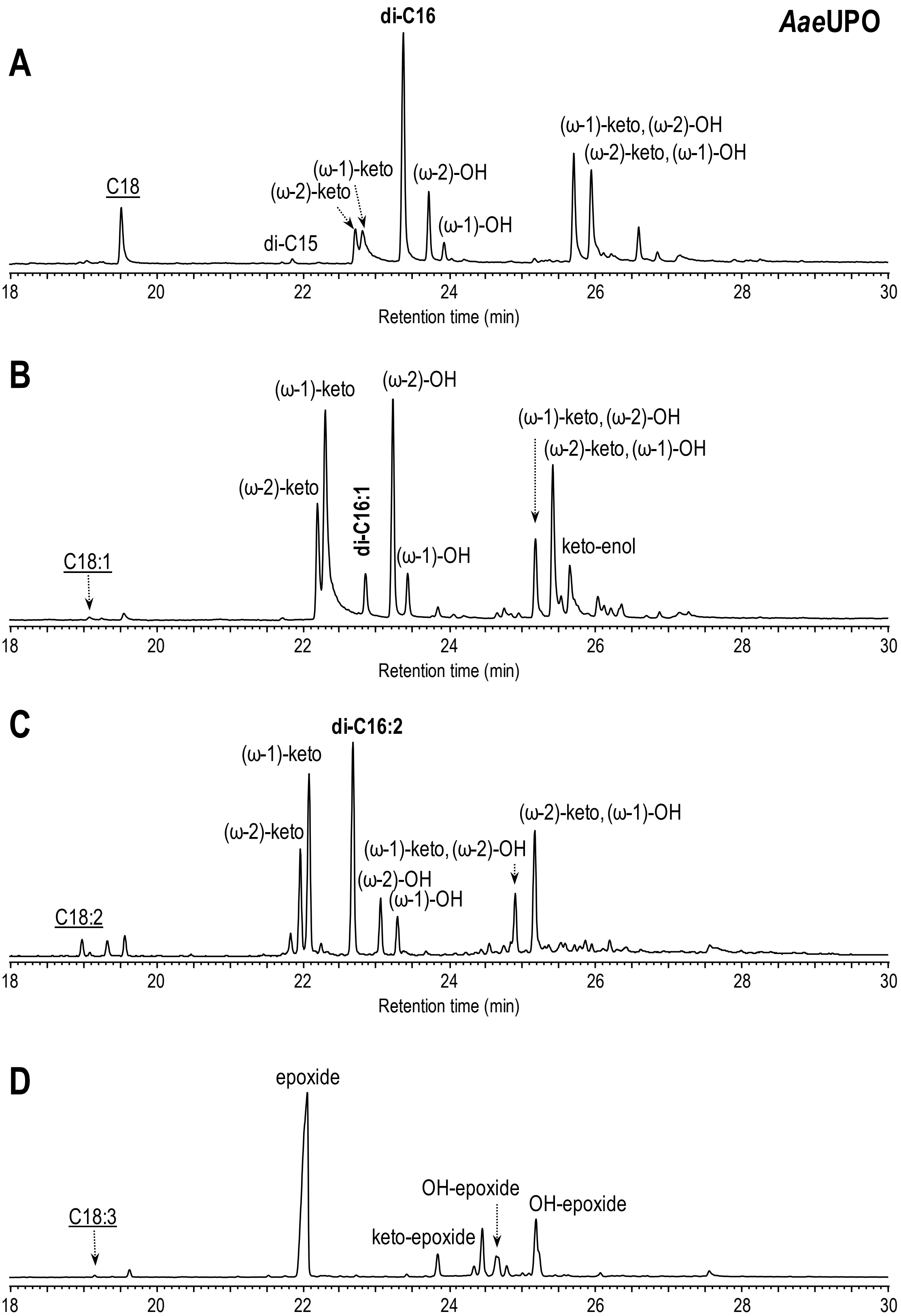

Figure S2. GC-MS analysis of *Aae*UPO reactions with 0.1 mM stearic (**A**), oleic (**B**), linoleic (**C**) and linolenic (**D**) acids (underlined) showing the dicarboxylic acids (in bold) together with the hydroxy, keto, enol and epoxide derivatives. Reaction conditions: A (1 μM enzyme, 20 mM H_2O_2, 25 h); B (0.4 μM enzyme, 5 mM H_2O_2, 1 h) and C, D (0.2 μM enzyme, 2.5 mM H_2O_2, 0.5 h).

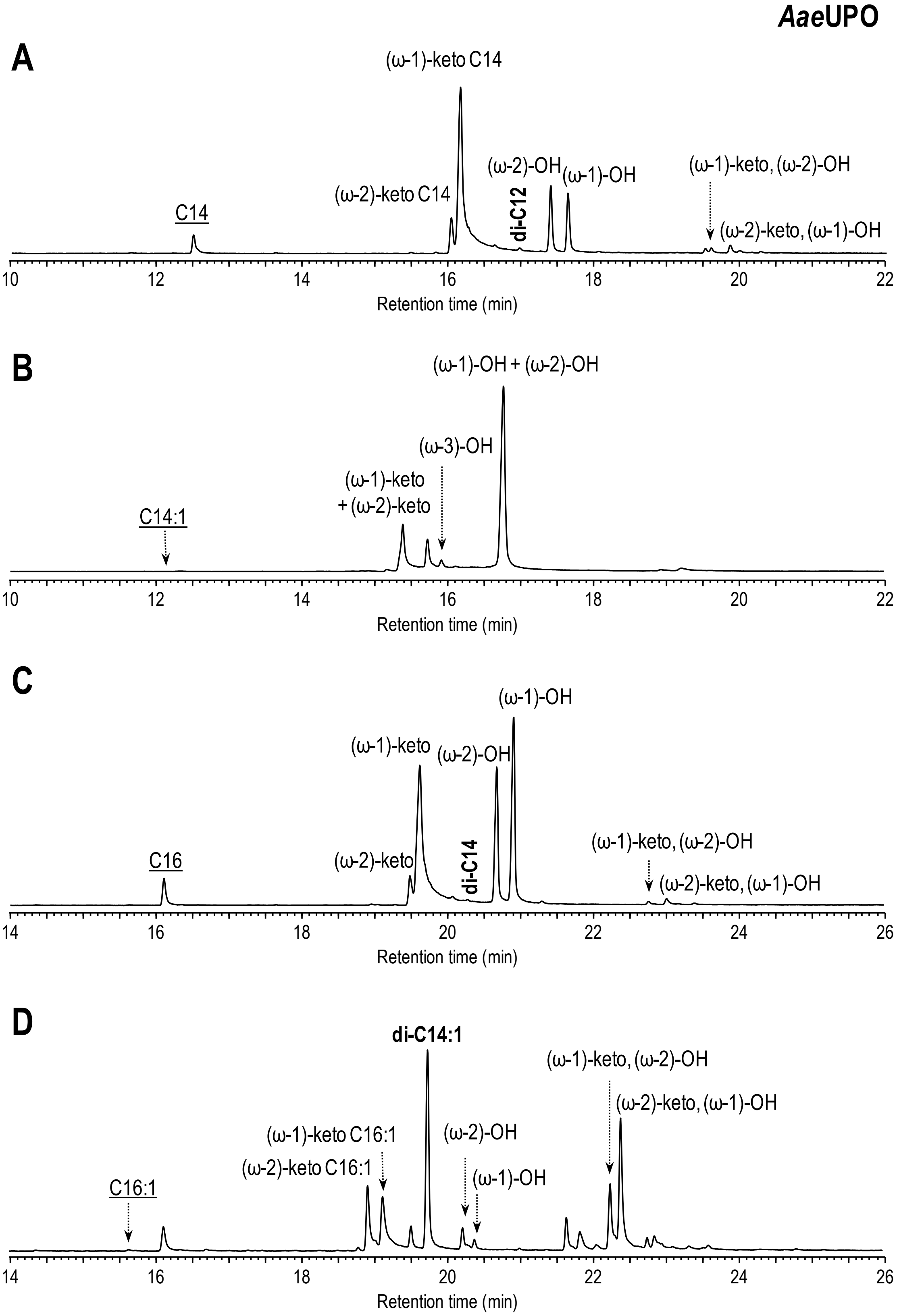

Figure S3. GC-MS analysis of *Aae*UPO reactions with myristic (**A**), myristoleic (**B**), palmitic (**C**) and palmitoleic (**D**) acids (underlined) showing the shortened dicarboxylic acids (in bold) together with

the hydroxy, keto, and enol derivatives of the substrate. Reaction conditions: A (0.1 mM substrate, 0.4 μM enzyme, 5 mM H_2O_2, 1 h, 20% acetone); B (0.1 mM substrate, 0.2 μM enzyme, 2.5 mM H_2O_2, 0.5 h, 20% acetone); C (0.1 mM substrate, 0.8 μM enzyme, 15 mM H_2O_2, 3 h, 40% acetone) and D (0.1 mM substrate, 0.8 μM enzyme, 15 mM H_2O_2, 3 h, 20% acetone).

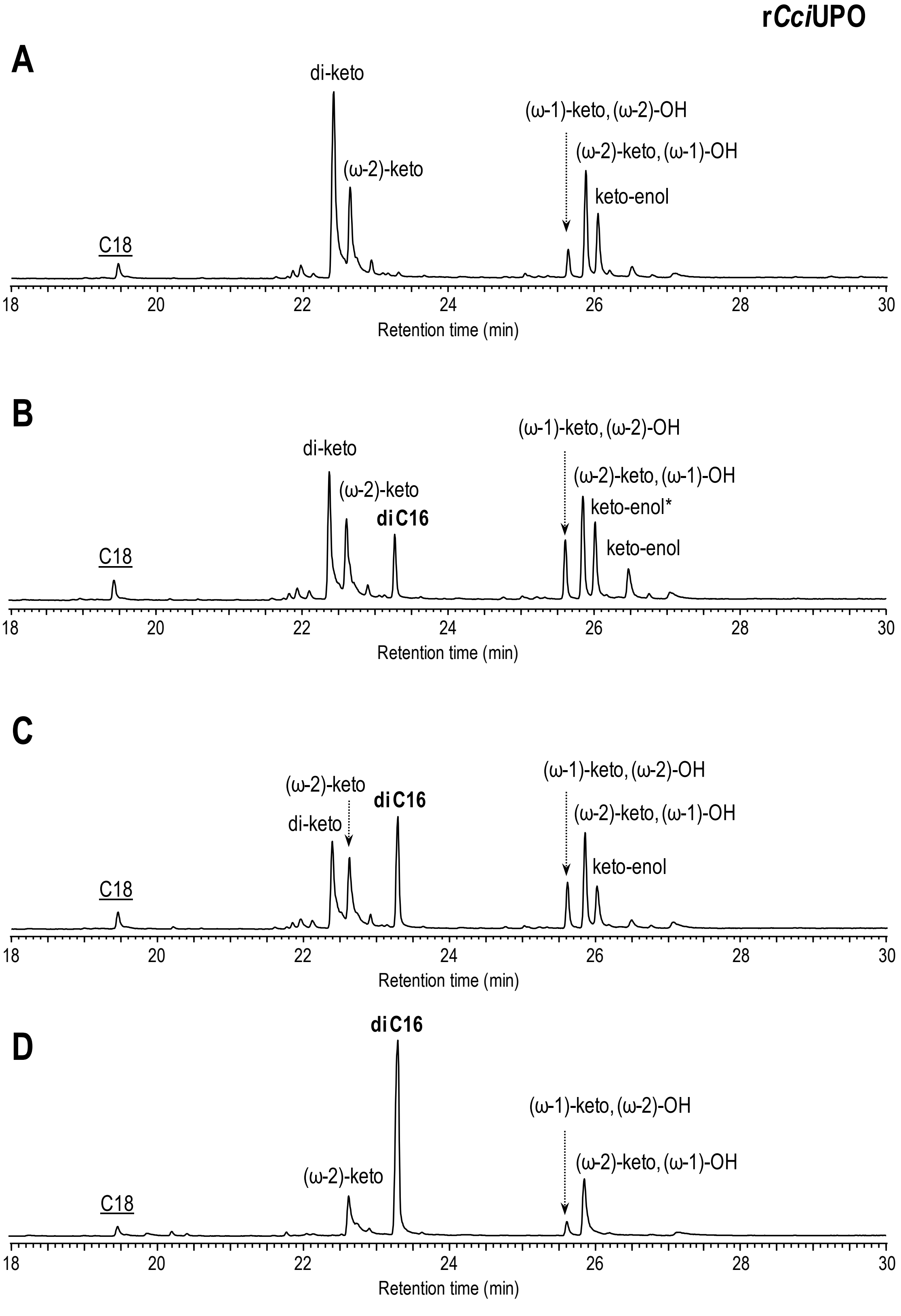

Figure S4. GC-MS of the reaction of 1.2 μM r*Cci*UPO with 0.1 mM stearic acid (underlined), after 3 h and 10 mM H_2O_2 (**A**). GC-MS of the products of the reaction A after addition of 1 mM (**B**), 3 mM (**C**) and 5 mM (**D**) H_2O_2 during 2 h in the absence of any enzyme. Higher doses of H_2O_2 (up to 15

mM) did not show differences respect to D. Solvent (acetone) was added in a 40% of the total volume in all cases. The 2C shorter dicarboxylic acid (in bold) is shown together with the hydroxy, keto and enol derivatives. Peak with asterisk was tentatively identified as keto-enol isomer.
